# Anti-Typhoid Inoculation

**Published:** 1913-08

**Authors:** 


ANTI-TYPHOID INOCULATION.—Among the many interesting and important topics discussed at the recent—May 5-6—annual meeting of the American Therapeutic Society held in Washington, D. C., none were of more general importance, nor deserving of more consideration than anti-typhoid inoculation as a preventive of typhoid fever; and yet, if the report of the proceedings of the Society published in the *Medical Record* of June 14, 1913, is a reflex of the attention accorded this important subject, few received less attention. The following rescript contains all, seemingly, that was said and done about it:

"Dr. F. F. Russel, Medical Corps, U. S. A., summarized the effects of anti-typhoid vaccination up to the close of the year 1912 in the United States army, both at home and abroad, among the officers and enlisted men. During the last four years approximately 200,000 persons had been immunized, without fatalities or untoward results. The custom at present was to revaccinate at the beginning of each four-year period of enlistment, not because all immunity had disappeared, but because it seemed unwise to trust to anything than the maximum immunity obtainable. Anti-typhoid vaccination had been a pronounced success in the naval and military services, in hospitals, schools, institutions, among pleasure seekers, and in contractors, especially those located on water-sheds; in fact, everywhere in civil and military life where it had been used. Its more general use, especially among the young, was advisable. The fact that it occasionally failed to afford complete protection was not a valid objection to its use, but rather an indication for its repetition, at intervals to be determined upon in the future. Its extended use would hasten the time when typhoid fever would become a negligible factor in our public health problems."

The experience of Dr. Russel, as epitomized in the excerpt, seems consonant with that of European army surgeons, especially those of the English and German armies, and should at once challenge the attention of civilian health officers throughout the world; and proper provision should be made for its *enforced* general application much as is done in vaccination against smallpox. With this difference, both inoculations should be obligatory, and heavy penalties exacted of the parents of those who fail to protect their families in these matters. No excuse whatever should be accepted; for which reason the inoculations should be made under direction of health officers, and should be free—"without money and without price"—and should be repeated as often as experience shall demonstrate to be necessary. In this way, and in this way alone—according to the lights before us—will smallpox and typhoid fever ever become negligible factors in the health problems that confront us. BIBB.

---

THE LEGISLATURE (now in session) provides $65,000 for a nurses' home at Galveston in connection with the Sealy Hospital, the Sealys to expend $80,000 in additions to the hospital. * * * The State Board of Health $60,000 one year; $48,000 the second year. The total appropriation for running the State two years is fourteen million odd dollars.

